# Update in Neurocritical Care: a summary of the 2018 Paris international conference of the French Society of Intensive Care

**DOI:** 10.1186/s13613-019-0523-x

**Published:** 2019-04-16

**Authors:** Mauro Oddo, Serge Bracard, Alain Cariou, Gérald Chanques, Giuseppe Citerio, Béatrix Clerckx, Bertrand Godeau, Anne Godier, Janneke Horn, Samir Jaber, Boris Jung, Khaldoun Kuteifan, Marc Leone, Alexandra Mailles, Mikael Mazighi, Bruno Mégarbane, Hervé Outin, Louis Puybasset, Tarek Sharshar, Claudio Sandroni, Romain Sonneville, Nicolas Weiss, Fabio Silvio Taccone

**Affiliations:** 1 Department of Intensive Care Medicine, CHUV-Lausanne University Hospital, Lausanne, Switzerland; 2 Department of Diagnostic and Interventional Neuroradiology, University of Lorraine and University Hospital of Nancy, Nancy, France; 3 Medical Intensive Care Unit, Cochin Hospital, Université Paris Descartes, Paris, France; 4 Department of Anaesthesia and Intensive Care, Montpellier Saint Eloi University Hospital, and PhyMedExp, University of Montpellier, INSERM, CNRS, 34295 Montpellier Cedex 5, France; 5 School of Medicine and Surgery, University of Milan-Bicocca, Milan, Italy; 6 Department of Intensive Care Medicine, University Hospitals Leuven, Louvain, Belgium; 7 Service de Médecine Interne, Centre de Référence des Cytopénies Auto-Immunes de l’Adulte, Hôpital Henri-Mondor, Créteil, France; 8 Fondation Adolphe de Rothschild, Department of Anesthesiology and Intensive Care, Paris Descartes University, Paris, France; 9 Department of Intensive Care, Academic Medical Center, University of Amsterdam, Amsterdam, The Netherlands; 10 Medical Intensive Care Unit, Montpellier Teaching Hospital, PhyMedex, University of Montpellier, Montpellier, France; 11 Service de Réanimation Médicale, GHRSMA, 68100 Mulhouse, France; 12 Service d’Anesthésie et de Réanimation, Hôpital Nord, Assistance Publique Hôpitaux de Marseille, Aix Marseille Université, Marseille, France; 13 ESGIB, ESCMID Study Group for Infectious Diseases of the Brain, Santé Publique France, 12, rue du Val-d’Osne, 94415 Saint-Maurice Cedex, France; 14 Department of Diagnostic and Interventional Neuroradiology, Rothschild Foundation, Paris, France; 15 Department of Medical and Toxicological Critical Care, Lariboisière Hospital, Paris, France; 16 Service de Réanimation Médico-Chirurgicale, CHI de Poissy-Saint Germain en Laye, Poissy, France; 17 Department of Anesthesia and Intensive Care, Pitié-Salpetrière Hospital, Paris, France; 18 Medical and Surgical Neurointensive Care Centre, Hospital Sainte Anne, Paris, France; 19 Istituto Anestesiologia e Rianimazione Università Cattolica del Sacro Cuore, Fondazione Policlinico Universitario Agostino Gemelli IRCCS, Rome, Italy; 20 Department of Intensive Care Medicine and Infectious Diseases, Hôpital Bichat-Claude, Université Paris Diderot, Paris, France; 21 Neurocritical Care Unit, Department of Neurology, Assistance Publique – Hôpitaux de Paris, La Pitié-Salpêtrière University Hospital, Sorbonne Université, Paris, France; 22 Department of Intensive Care, Erasme Hospital, Université Libre de Bruxelles (ULB), Route de Lennik, 808, 1070 Brussels, Belgium

**Keywords:** Neurocritical care, Neurointensive care, Expert review, Update, Brain injury, Delirium, Coma

## Abstract

The 2018 Paris Intensive Care symposium entitled “Update in Neurocritical Care” was organized in Paris, June 21–22, 2018, under the auspices of the French Intensive Care Society. This 2-day post-graduate educational symposium comprised several chapters, aiming first to provide all-board intensivists with current standards for the clinical assessment of altered consciousness states (including coma and delirium) and peripheral nervous system in critically ill patients, monitoring of brain function (specifically, electro-encephalography) and best practices for sedation—analgesia—delirium management. An update on the treatment of specific severe brain pathologies—including ischaemic/haemorrhagic stroke, cerebral venous thrombosis, hypoxic-ischaemic brain injury, immune-mediated and infectious encephalitis and refractory status epilepticus—was also provided. Finally, we discuss how to approach some difficult decisions, namely the role of decompressive craniectomy and prognostication models in patients with head injury. For each chapter, the scope of the present review was to provide important issues and key messages, provide most recent and relevant literature in the field, and briefly describe new developments in the field.

## Introduction

The 2018 Paris Intensive Care (PIC) symposium organized in Paris, June 21–22, 2018, under the auspices of the French Intensive Care Society focused on neurocritical care. This 2-day educational symposium provided general intensivists with an overall view of basic principles of neurological assessment, neuro-monitoring and sedation management to apply at the bedside when caring for the injured brain. Specific severe acute brain pathologies were reviewed aiming at providing an update on current best practice care of several diseases—such as ischaemic/haemorrhagic stroke, cerebral venous thrombosis, hypoxic-ischaemic brain injury, immune-mediated and infectious encephalitis and refractory status epilepticus. We also discussed difficult decisions such as the role of decompressive craniectomy and neuro-prognostication following head injury. The objective of the present review is to summarize, for each chapter, the most important issues and key messages (*what is important*), and briefly describe new developments in the field (*what is new*), aiming at providing updated relevant literature in the field.

## Caring for the injured brain: general aspects

See also Table 1.

**Table 1:** Care of the injured brain in the general ICU: basic comprehensive aspects

Topic	What is important	What is new
Management of the comatose patient	GCS, brainstem reflexes, FOUR score Brain imaging (CT scan, MRI) Multimodal monitoring (ICP, brain oximetry, TCD)	Automated infrared pupillometry q-EEG Multimodal monitoring-based individualized therapy
Management of the delirious patient	CAM-ICU, ICDSC scores Minimized targeted sedation Early active mobilization	ABCDEF, e-CASH bundles Alternative sedatives (dexmedetomidine) Prophylactic use of anti-psychotics not effective
Management of ICU-acquired weakness	MRC muscle scale, ENMG Optimize nutrition and glycaemic control Early active mobilization	Assessment of diaphragmatic dysfunction Physical (electrical) therapy Mobilization protocols

### Assessment of the comatose patient

#### What is important

Assessment of the comatose patient relies on clinical examination, neuroimaging and neuro-monitoring. Neurological examination includes the Glasgow Coma Scale (GCS)—and particularly the GCS-motor response (GCS-M) to pain—and brainstem reflexes, with a specific attention to pupillary aspect (symmetry) and functionality (reactivity) [1]. The Full Outline of Unresponsiveness (FOUR) score comprises the assessment of both the cortical and brainstem functions [2].

Neuroimaging encompasses (a) brain CT scan, for diagnosis of acute thromboembolic and haemorrhagic complications, hydrocephalus, oedema, abscess; (b) transcranial Doppler ultrasound to estimate cerebral perfusion and intracranial compliance non-invasively [3]; (c) magnetic resonance imaging (MRI) imaging to quantify the extent and location of brain damage and for outcome prognostication [4]. Neuro-monitoring includes several invasive (intracranial pressure, brain oximetry) and non-invasive modalities (transcranial doppler, electro-encephalography [EEG], automated infrared pupillometry). Indications and optimal combination of monitoring modalities is dependent on injury type and severity, and the expected risk of secondary cerebral damage.

#### What is new

Automated infrared pupillometry enables the quantitative assessment of basic fundamental neurological tests, such as pupillary symmetry and reactivity [5–7]. Multimodal monitoring has become central for individualized targeted neurocritical care, focused on improving altered brain physiology at the bedside [8] and for neuro-prognostication [9, 10].

### EEG monitoring of brain function in the comatose patient

#### What is important

Continuous EEG (c-EEG) is indicated for the management of refractory status epilepticus. In this setting, when to use continuous versus intermittent EEG remains debated [11]. Most epileptic abnormalities can be captured using a 2-h recording [12], while c-EEG for 24 h or more may be indicated in high-risk patients (comatose and prior seizures) [13]. C-EEG may be part of multimodal ICU monitoring, e.g. to monitor sedation depth and pharmacological burst-suppression, to detect secondary ischaemia, or for coma prognostication [14, 15]. There are several barriers to cEEG implementation, including the requirement for continuous access to technicians and neurophysiologists [15] and difficulties in data storage. Also, there is a lack of consensus on the clinical significance of selected outcome predictive EEG patterns that should be prioritized in the ICU setting, i.e. non-convulsive seizures, hemispheric asymmetry due to evolving ischaemic conditions, and sedation-induced EEG suppression. Finally, lack of standardization and reliability, particularly with respect to reactivity to pain [16], and the need for an international consensus to define the main EEG prognostic features [17] are some of the still unsolved questions related to the use of EEG in critically ill in comatose patients.

#### What is new

Spectral analysis (quantitative EEG) and software enabling artefact elimination facilitate availability of EEG at the bedside and reduce the risk of reading errors and false interpretation. EEG training courses improve the accuracy of quantitative EEG reading by general intensivists, raising hope for future effective implementation in daily ICU practice [18, 19].

### Assessment of delirious patient

#### What is important

Brain dysfunction in the ICU patient goes beyond *comatose states* and includes a wide spectrum of consciousness disorders that characterize *delirium states*. Delirium is defined by a disturbance in attention and awareness developing over a short time period, fluctuates during the day, and is accompanied by a change in cognition [20]. Delirium assessment needs first evaluating the level of sedation, e.g. by the Richmond Agitation Sedation Scale (RASS), and then utilizing delirium assessment scales such as the Confusion Assessment Method for the ICU (CAM-ICU) or the Intensive Care Delirium Screening checklist (ICDSC) [21]. Delirium pathophysiology is complex, resulting from hypoxic, inflammatory and metabolic insults, and may be aggravated or amplified by multiple factors. These include *non*-*modifiable* factors (age, co-morbidities, pre-existing cognitive impairment and psychoactive drug use, severity of illness) and *potentially modifiable* factors (renal failure, hypernatremia, hypercapnia, dysglycemia, sedatives, neurotoxic antimicrobial agents, immobilization and use of physical restraints) [22, 23].

#### What is new

Emerging evidence established a link between delirium and brainstem dysfunction, and particularly autonomic nervous system dysfunction and impairment of cholinergic activity [24]. This correlates clinically with the abolition of cough reflex and worse outcome seen in severe critically ill patients requiring deep sedation [25]. Accurate evaluation of brainstem function, and the development of new scores such as the Brainstem Reflexes Assessment Sedation Scale (BRASS) [26], or the use of automated pupillometry for quantitative assessment of pupillary function [5], may improve delirium assessment and outcome prediction. EEG may be helpful for differential diagnosis and investigating potential contributing factors (over-sedation, antibiotic toxicity), as well as in predicting prognosis [27, 28].

### Sedative strategies and delirium management

#### Sedative strategies

##### What is important

The general trend in the ICU about sedation is “less is more”. Strategies aimed at minimizing sedation include [29]:

1. Daily interruption of sedation, or at least, a daily consideration for sedation interruption;
2. Use of sedation algorithms, to avoid over-sedation;
3. Analgesia prioritization-based algorithm;
4. Patient-centred care based on the assessment and treatment of specific symptoms (pain, anxiety, delusion), to avoid deep sedation [30].

##### What is new

Sedative-induced delirium was the most frequent delirium subtype in a recent study, and the subtype for which prolonged duration of delirium was associated with the greatest impact on long-term cognitive dysfunction [31]. A strategy of immediate interruption of sedation, in ICU patients after major abdominal surgery (mostly with septic shock), translated into reduced delirium incidence and hospital length of stay [32]. A strategy based on minimizing benzodiazepine is recommended by recent consensus documents [18]. Although this may reduce delirium risk in non-severe surgical ICU patients, new data showed that delirium prevention using anti-psychotics is ineffective in more severe critically ill patients [33]. Also, there is currently no data supporting the use of monitoring systems, such as the bispectral index, to tailor sedation in the critically ill. Continuous infusion of dexmedetomidine may accelerate delirium recovery [34], while intermittent nightly administration of low dose dexmedetomidine promotes sleep and reduces delirium [35]; however, this study suffered from a small sample size and requires confirmation in larger cohorts. In the AWARE randomized multicenter trial, all adult patients requiring mechanical ventilation for more than 48 h were randomized into two groups, based on standard sedation practices (control; *n* = 590) versus a sedation strategy according to an algorithm which provided a gradual multilevel response to pain, agitation, and ventilator dyssynchrony and promotion of the use of alternatives to continuous infusion of midazolam or propofol (intervention; *n* = 584) [36]. Although the use of midazolam and propofol was significantly lower in the intervention group, 90-day mortality was lower, although not significantly singnificant (39 vs. 44% in the control group, *p* = 0.09), in the intervention group.

### Developing management approaches for delirium

#### What is important

Delirium management implies the implementation of a multistep delirium management bundle, using different approaches, such as the e-CASH and ABCDEF bundles [20, 37], which include:

1. Early detection and correction of risk factors (sepsis, metabolic and respiratory disturbances);
2. Control of iatrogenic risk factors (sedation; in particular, limiting or avoiding benzodiazepines);
3. Favouring non-pharmacological interventions to treat anxiety, pain and sleep privation in order to avoid the adverse events associated with psychoactive drugs;
4. Promoting early active mobilization.

##### What is new

A multiapproach delirium management bundle, coupled with targeted sedation, mechanical ventilation weaning strategies, and early mobilization, is effective in reducing delirium incidence, and even mortality [38, 39]. Whether non-pharmacological preventive strategies for anxiety, pain and sleep deprivation may benefit cognitive function needs further investigation [40]. In this setting, music therapy has gained recent interest in the area of critical care [41]. Artificial light is ineffective to reduce delirium in the ICU [42].

### ICU-acquired weakness

#### Pathophysiology

##### What is important?

ICU-acquired weakness (ICU-AW; defined as a generalized hypotonic and symmetrical weakness, with sparing of facial muscles, occurring during and/or after the ICU stay) is a common and serious complication of critical illness [43]. The pathophysiology is multifactorial [44], but include severe underlying disease, sepsis and/or multiorgan failure, and older age. Bed rest may also be an important factor, since it causes a significant decrease in strength and cardiorespiratory endurance.

##### What is new?

Animal models are lacking or may fail to clarify ICU-AW pathophysiology [45, 46]. Clinical research on ICU-AW during the early phase may be limited by coagulation disorders that often hamper neurophysiological testing and biopsies Although inflammation is consistently increased in experimental and clinical studies, infiltration of inflammatory cells into the neural and muscular tissue is rarely found [47]. Blood glucose control with insulin, targeting normoglycemia, and early weaning from mechanical ventilation may attenuate ICU-AW [48].

#### Clinical aspects

##### What is important

The Medical Research Council (MRC) muscle scale is recommended for the diagnosis of ICU-AW, with a score below 48 defining ICU-AW [49]. Physical examination should be completed by electrophysiological (electroneuromyography, ENMG), imaging and biology tests to rule out alternative diagnoses, in particular when the clinical picture involves asymmetric weakness, pyramidal syndrome, facial involvement, ascending/descending weakness, autonomic nervous system dysfunction and extra-neurological signs [50].

##### What is new

The diaphragm is more susceptible to systemic inflammation than limb muscles and its structure and function is rapidly altered after only 36 h of mechanical ventilation in humans [51, 52]. ICU-AW and diaphragmatic dysfunction share the same risk factors; diaphragmatic dysfunction affects the weaning process from the ventilator but also survival [53, 54]. ICU-AW and diaphragmatic dysfunction have an impact on long-term morbidity and mortality. Innovative clinical trials are needed to examine the effectiveness of early bundle strategies that may include electrical stimulation, physical therapy and optimized nutrition for preventing or reducing the occurrence of ICU-AW and diaphragmatic dysfunction.

#### Early mobilization

##### What is important

Early mobilization and physical activity is effective on the short-term in several studies [39, 55], although results of long-term outcome studies are more controversial [56, 57]. The detrimental physiological effects of restricted mobility on all systems, and the benefits of being upright and moving have been widely reported. Issues related to early physical activity and mobilization of patients in the ICU as a therapeutic option including safety, therapy duration and intensity, and implementation have only recently been a shared focus of interest in the ICU. Accurate assessment of cardiorespiratory reserve and rigorous screening for other factors that could preclude early mobilization is of paramount importance [58].

##### What is new

Different (modifiable) barriers for mobilization and rehabilitation were identified by nurses, physiotherapists and physicians: they include lack of staff and supporting equipment, no protocol, no mobility culture, lack of planning and coordination, no ‘champion’ in the team, or ‘standing bed rest’ order. A mobilization protocol, consisting of six levels of early mobilization and physical activity (using objective measurements including ‘basic’ assessment, adequacy score, muscle strength and functional performance) could help solving such barriers [59].

In a single-center randomized clinical trial enrolling more than 300 patients, the addition of early in-bed leg cycling plus electrical muscular stimulation (quadriceps muscles) to a strategy of standardized early rehabilitation (i.e. a weekday progressive multistep program beginning with 10 passive range of motion exercises with each limb joint applied once every weekday by physiotherapists to comatose or sedated patients, followed by passive or active exercises and then fully active muscle exercises (i.e. transfer to the edge of the bed or to a chair, standing, and walking) did not improve global muscle strength at ICU discharge [60].

## Caring for the injured brain: management of specific pathologies

See also Table 2.

**Table 2:** Caring for the injured brain: specific management of severe acute cerebral pathologies

Disease	What is important	What is new
Hypoxic-ischaemic brain injury	In-hospital targeted temperature management (TTM) Optimize blood pressure and ventilatory management (SaO_2_, CO_2_) Multimodal neuro-prognostication	Pre-hospital TTM not effective Use automated devices for TTM Precise temperature target undefined Quantitative tools (pupillometry, MRI) improve neuro-prognostication
Immune-mediated encephalitis	≈ 30% of encephalitis are of non-infectious origin Anti-NMDAR encephalitis most common form	Two main patterns in the ICU: (1) anti-NMDA-R encephalitis (psychiatric symptoms, seizures and abnormal movements) (2), anti-NMDA, GABA-A or LGI-1-R (refractory status epilepticus)
CNS vasculitis	Two main forms: Primary (primary CNS angitis, PACNS) or Secondary to systemic diseases (infections, autoimmune vasculitis with or without anti-cytoplasmic antibodies (ANCA), connective tissue diseases, malignancies, lymphoma) MRI is essential to diagnosis	Treatment of CNS vasculitis requires high-dose of steroids; cyclophosphamide and rituximab may be added (no consensus)
Refractory status epilepticus	Maintain general anaesthesia for at least 24 h Continuous EEG monitoring	Ketamine is an alternative to barbiturates Novel anti-epileptic drugs available (levetiracetam, brivaracetam, lacosamide, perampanel, etc.)
Ischaemic stroke	Mechanical recanalization and alteplase Therapeutic time window can be extended beyond 12 h	Tenecteplase as alternative to alteplase
Anticoagulation-associated intracerebral haemorrhage	Rapid reversal with the use of PCC	Idarucizumab for dabigatran reversal Andexanet-alpha for reversal of other direct oral anticoagulants (available in the US only)
Cerebral venous thrombosis	Early anticoagulation with heparin	Endovascular therapy and/or decompressive craniectomy for severe forms Favourable prognosis in the majority of cases if early intervention is applied
Delayed ischaemia after subarachnoid haemorrhage	Additional mechanisms other than vasospasm play a role Diagnosis based on the combination of clinical, and neuroimaging data Nimodipine prophylaxis Management based on the combination of medical (BP augmentation) and endovascular (local vasodilatory drugs ± angioplasty) therapies	MMM may help in the diagnosis in comatose patients
TBI surgical management	Secondary decompressive craniectomy may increase dependency in survivors	Individualized multidisciplinary decisions are recommended
TBI prognosis	IMPACT and CRASH scores	Advanced MRI diffusion at least 1 week after injury (DWI and DTI)

### Hypoxic-ischaemic brain injury (HIBI)

#### What is important

Currently, no pharmacological approach is available to treat HIBI [61] and the only recommended intervention with proven efficacy in comatose adults after out-of-hospital cardiac arrest with an initial shockable rhythm is targeted temperature management (TTM), started immediately on patient arrival to the hospital [62]. Pre-hospital cooling, using rapid infusion of large volumes of cold intravenous fluid, is not recommended. Modern devices with automated temperature retro-feedback are preferred to keep constant temperature, although there is no specific recommendation for a precise technique. Neuroprotective strategies include maintenance of adequate brain perfusion—by optimizing systemic hemodynamics, blood pressure and keeping normal PaCO_2_—and oxygenation, by using controlled oxygenation (SaO_2_ 94–98%) and avoiding hyperoxia [63]. Neuro-prognostication is based on multimodal assessment, including neurological tests (pupillary reactivity) and electro-physiologic assessment (mainly, somatosensory evoked potentials and EEG), with clinical decisions taken at least > 72 h, paying specific attention to exclude potential confounders (residual sedation, metabolic derangements) and to repeat the tests in case of discordant findings [10]. The most robust predictors include a bilateral absence of corneal and/or pupillary reflexes or N20 waves of short-latency somatosensory evoked potentials. These robust predictors have a high specificity, but their sensitivity rarely exceeds 50%. If results of most robust predictors are normal, a second set of predictors can be tested, such as high blood levels of neuron specific enolase (NSE), presence of malignant EEG patterns (status epilepticus, burst-suppression, non-reactive background), and signs of diffuse HIBI on brain CT or MRI.

#### What is new

The ongoing international multicentre randomized controlled TTM-2 trial will compare two target temperature strategies, i.e. 33 °C versus fever control (< 37.8 °C) in a large group (*n* = 1900) of comatose cardiac arrest patients and should provide additional robust data to available evidence. In recent years, new progress towards a better standardization of neuro-prognostic indices has been accumulating. Studies adopting the 2013 American Clinical Neurophysiology Society guidelines to define malignant EEG patterns showed that these patterns are not only reproducible with acceptable interrater agreement, but also predict early with both high sensitivity and specificity [17]. Automated quantitative pupillometry achieved higher sensitivity and specificity than conventional pupillary assessment to assess poor prognosis [7, 64]; in particular, a neurological pupil index (NPI) < 2 at 24 h after arrest had a specificity of 100% to identify patients with unfavourable neurological outcome [64]. In patients with prolonged (≥ 7 days) unconsciousness after cardiac arrest, measurement of fractional anisotropy of the white matter of the brain using diffuse tensor MRI imaging, predicted poor neurological outcome with 100% specificity and 89% sensitivity [65].

### Immune-mediated encephalitis

#### What is important

Up to one third of encephalitis is of non-infectious origin [66, 67], and in one study 21% of patients with presumed infectious encephalitis actually had immune-mediated encephalitis [66]. Among immune-mediated encephalitis, anti-*N*-methyl-d-aspartate receptor (NMDA-R) encephalitis is the most commonly encountered form. The relevant antibody can be detected in the blood and/or CSF but, despite extensive CSF, EEG and MRI work-up, immune-mediated encephalitis without identified antibodies still exist. First-line therapy includes high-dose steroids and immunotherapy (IV immunoglobulins or plasma exchange). Second-line therapy (rituximab or cyclophosphamide) is started in the absence of rapid neurological improvement [68, 69]. Careful search of neoplastic causes (e.g. ovarian teratoma) is mandatory, because surgical removal is indicated in this case [70].

#### What is new

The recent description of patients with a common clinical presentation in association with ovarian teratoma and the identification of specific antibodies directed against *N*-methyl-d-aspartate receptor (NMDA-R) changed our view of encephalitis [70]. Since, several new antibodies associated to encephalitis are described each year [70]. Diagnostic criteria are now available [70]. In the ICU, two main patterns are encountered: (1) encephalitis with psychiatric symptoms, seizures and abnormal movements, most frequently associated with anti-NMDA-R antibodies, and (2) refractory status epilepticus, associated with anti-NMDA-R, GABA-A or LGI-1 antibodies. Importantly, prognosis is good in the majority of patients [68].

### Central nervous system vasculitis

#### What is important

CNS vasculitis causes inflammation and destruction of blood vessel walls affecting brain, spinal cord and/or meninges. It is rare (incidence 2.4/million per year) and is classified as primary (primary CNS angitis, PACNS) or secondary to systemic diseases, including infections (bacteria, viruses, parasites and fungi), vasculitis with or without the presence of anti-cytoplasmic antibodies (ANCA), connective tissue diseases (particularly systemic lupus erythematosus and Sjögren disease) and malignancies, including cancers and lymphoma. Clinical manifestations are non-specific and heterogeneous (headache, focal motor or sensory abnormalities, cognitive impairment, seizures), and clinical presentation can be acute with a feature of stroke involving multiple and bilateral vascular territories or chronic and progressive with cognitive deficit and psychiatric manifestations. CSF reveals aseptic meningitis with modest increased cellularity and it may be normal in 20% of cases. MRI is essential to diagnosis and reveals multiple ischaemic lesions or haemorrhages. A normal MRI associated with normal CSF analysis exclude the diagnosis of CNS vasculitis. Infectious, neoplastic and autoimmune conditions should be ruled out.

#### What is new

The treatment of CNS vasculitis is an emergency and should be related to aetiology. High dose of steroids is required in PACNS and for some groups, cyclophosphamide should be associated although there is no consensus [71, 72]. The indication of antiplatelet agents is not clear and the interest of rituximab and other emergent biotherapies is not demonstrated. To conclude, unlike the progress of neuroimaging, the diagnosis of CNS remains a challenge requiring multimodal approach and a close cooperation between specialists (i.e. neurologists, neuroradiologists, immunologists…).

### Refractory status epilepticus

#### What is important

Management of status epilepticus (SE) is based on four immediate simultaneous actions [71, 74]:

1. Confirm diagnosis, eliminate non-epileptic psychogenic events, encephalopathies with myoclonus and other abnormal movements: video-EEG may help in this setting.
2. Treat systemic consequences of SE and other factors of cerebral aggression, consequences of treatment, and complications of intensive care.
3. Treat aggravating factors (e.g. fever).
4. Ensure sustained interruption of epileptic activity:
  - 0–5 min: benzodiazepines, renewed if seizures last more than 5 min; clonazepam is an effective alternative to lorazepam and in the absence of venous access, intramuscular midazolam is an appropriate option.
  - > 5 min: start an anti-epileptic drug: fosphenytoine, valproate (contraindicated in women of childbearing age), phenobarbital, or levetiracetam; defining the optimal treatment sequence is debated [75].
  - > 20–30 min: SE persisting after two appropriately selected and dosed parenteral medications including a benzodiazepine is defined as refractory SE (RSE). Coma induction with midazolam, propofol then pentobarbital/thiopental—and increasingly with ketamine—is recommended [76]. Seizures must be clinically unquestionable or EEG-proven (non-convulsive status post-generalized status); therefore, EEG is an integral part of the management of RSE. Guidelines recommend obtaining the suppression of electrographic seizures or aiming for burst-suppression for at least 24 h before gradual reduction in IV anaesthetics [73].

SE persisting for > 24 h after the onset of general anaesthesia is defined as super-refractory SE (SRSE) [76]. The treatment remains empiric. An immunological cause must be ruled out and treated early. Although SRSE has a poor prognosis, some patients may recover even in prolonged cases. Advice by and/or transfer to centres with specific neurology expertise are advisable [77].

#### What is new

Novel anti-seizure medications (e.g. levetiracetam, brivaracetam, lacosamide, perampanel), with a better safety and pharmacokinetic profile, hold promise for the treatment of RSE. Further studies are required to clarify the indications and optimal use of such novel agents [78]. While avoidance of fever is recommended in RSE, therapeutic hypothermia (32–34 °C for 24 h) does not confer any additional benefit [79].

### Acute ischaemic stroke

#### What is important

Mechanical thrombectomy with intravenous alteplase (rtPA) is the current gold standard to improve neurological outcomes of acute ischaemic stroke consecutive to large vessel occlusion of the anterior circulation (i.e. internal carotid and middle cerebral arteries) based on six randomized controlled trials showing the superiority of such strategy over rtPA alone (number needed to treat 2.6) [80]. The benefit of mechanical thrombectomy was the most important for the oldest patient over 80 years old and those with the most severe strokes. The therapeutic window for mechanical thrombectomy initially established at 6 h after symptoms onset has moved to 24 h since the recent publication of two randomized trials showing the benefit of endovascular therapy in highly selected patients with multimodal imaging [81].

#### What is new

Trials are currently ongoing to address the benefit of mechanical thrombectomy alone versus mechanical thrombectomy in association with intravenous alteplase. Tenecteplase may be associated with higher recanalization rates compared to alteplase in patients with large vessel occlusions [82, 83]. The best strategy for patient transfer (i.e. “drip and ship” thrombolysis at a local stroke unit and transfer to a comprehensive stroke centre for mechanical thrombectomy versus “mother ship” direct transfer to the comprehensive stroke centre) remains to be established.

### Management of acute ischaemic stroke in patients treated with oral anticoagulants

Systemic thrombolysis with rtPA is contra-indicated in patients treated with anticoagulants, including direct oral anticoagulants (DOAC; including dabigatran, rivaroxaban, apixaban, and edoxaban). Idarucizumab, the specific antidote of dabigatran, induces immediate normalization of coagulation, without intrinsic thrombotic effect. Based on expert consensus, the use of rtPA is proposed for dabigatran-treated patients facing ischaemic stroke immediately after dabigatran reversal [84]. Rapid measurement of dabigatran concentration may improve the selection of patients that may benefit from reversal. Such strategy is not recommended with other DOAC.

### Anticoagulant associated brain haemorrhage

#### What is important

Spontaneous oral anticoagulation-related intracerebral haemorrhage is associated with larger haematoma volumes and increased rates of haematoma enlargement, leading to higher mortality rates. Therefore, prevention of secondary haematoma expansion is an important goal, based on urgent reversal of anticoagulation. Whereas appropriate reversal of vitamin K antagonists (VKA) is associated with a decrease in mortality, whether reversal of DOAC may improve outcome has never been demonstrated [85].

#### What is new

Management of DOAC reversal is evolving: activated or non-activated prothrombin concentrates were initially recommended, despite limited data on their safety and efficacy. Andexanet-alpha, the specific antidote to factor Xa-inhibitors, is not marketed yet in Europe and raises concerns regarding its potential thrombotic risk. Idarucizumab, the specific antidote of dabigatran, is currently available. In a mouse model of dabigatran-related ICH, idarucizumab not only prevented haematoma expansion but also reduced mortality [86]. Therefore, as for VKA, guidelines recommend urgent reversal in patients with DOAC-related ICH, irrespective of the agent [87]. The role of surgery remains controversial and haematoma removal should be reserved only for salvageable patients (i.e. young age with rapid deterioration) with clinical and/or CT signs of brain herniation.

### Cerebral venous thrombosis

#### What is important

Cerebral venous thrombosis is an uncommon cause of stroke (< 1% of all causes). At the acute phase, treatment includes management of the associated condition (infection, inflammatory conditions…), anticoagulation with low molecular weight or unfractionated heparin, treatment of intracranial hypertension, prevention of recurrent seizures and headache relief. Prognosis is generally good: mortality is below 5%, with only 15% of the patients remaining dependent [88].

#### What is new

In severe cases, decompressive surgery (i.e. hemicraniectomy and/or haematoma drainage) is lifesaving and often results in good functional outcome, irrespective of age, coma, aphasia, bilateral lesions, or non-reactive mydriasis [89]. Endovascular intervention is an alternative option for patients with severe forms on admission or with neurological deterioration despite the appropriate use of anticoagulation, especially in patients with thrombosis of the cerebral deep venous system and without large expanding hemispheric lesions [90]. The publication of the TOACT (Thrombolysis or anticoagulation for cerebral venous thrombosis) trial is expected; this randomized trial comparing endovascular treatment (thrombolysis with urokinase or rtPA and/or thrombectomy of any type) versus heparin will provide additional evidence on the best therapeutic strategy (clinical trials.gov NCT01204333).

### Delayed ischaemia after subarachnoid haemorrhage

#### What is important

About 30% of patients suffering from aneurysmal subarachnoid haemorrhage (SAH) develop delayed cerebral ischaemia (DCI), defined as the occurrence of focal neurological impairment (such as hemiparesis, aphasia, apraxia, hemianopia, or neglect), or a decrease of at least 2 points on the Glasgow Coma Scale, with an acute onset and not attributed to other surgical or medical conditions [91]. DCI pathophysiology is complex; although the presence of cerebral vasospasm is often associated with the occurrence of this condition, DCI may also result from microvascular dysfunction, neuro-inflammation, cortical spreading depolarization or an imbalance between vasoactive substances, in the absence of narrowing of large intracranial vessels. Importantly, DCI, but not cerebral vasospasm, is an independent determinant of morbidity and mortality in SAH patients. Oral nimodipine is recommended for prophylaxis after SAH, despite its effect is independent from the occurrence of vasospasm, it is supported by very old and questionable evidence, and there are no data on DCI.

#### What is new

Drugs, such as intravenous magnesium or endothelin-receptor antagonists, failed to provide any benefit on the neurological outcome of SAH patients, despite reducing vasospasm [92]. One limitation in the management of DCI is the lack of standardized neuro-monitoring for early detection and an appropriate therapy besides the use of vasopressors to increase mean arterial pressure. Concerning neuro-monitoring, the use of transcranial doppler (TCD) and continuous electro-encephalography (cEEG) in clinically evaluable patients may help to detect cerebral disturbances and immediately perform brain imaging (i.e. brain CT angiography, CTA—brain CT perfusion, CTP) to exclude the presence of clinically relevant cerebral vasospasm (i.e. resulting in brain hypoperfusion). In comatose patients, the combination of TCD/cEEG with brain tissue oxygen monitoring or microdialysis may effectively detect early DCI when clinical examination is unreliable [93, 94]. If DCI is related to cerebral vasospasm and vasopressor therapy does not improve clinical conditions or brain oxygenation, alternative interventions may include endovascular therapies (i.e. intra-arterial nimodipine or milrinone, balloon angioplasty, intra-carotid continuous infusion of vasodilators), often combined with systemic inotropic therapy.

### Clinical dilemmas: surgical decompression

#### What is important

Decompressive craniectomy (DC), i.e. surgical removal of a part of cranial vault with *dura mater* opening, is extremely effective in reducing brain herniation and intracranial pressure. This procedure is lifesaving, with controversial results on neurological recovery. In patients with large ischaemic stroke, DC decreases death rate and improves functional status. However, the proportion of patients with good neurological recovery remains low [95, 96]. In stroke patients aged < 60 years with unilateral MCA infarctions who deteriorate neurologically within 48 h despite medical therapy, DC reduces mortality by close to 50%, with 55% of the surgical survivors achieving moderate disability (able to walk) or better (modified Rankin scale, mRS score 2 or 3) and 18% achieving independence (mRS score 2) at 12 months, while for patients > 60 years of age DC reduces mortality by close to 50%, with 11% of the surgical survivors achieving moderate disability (able to walk [mRS score 3]) and none achieving independence (mRSscore ≤ 2) at 12 months. In two clinical trials on patients with severe head trauma, DC was effective in reducing mortality [97, 98]. However, the rate of head trauma patients with severe disability and/or vegetative state was significantly higher in the DC group, as compared to controls. However, both the studies above have limitations; one trial used only bi-frontal DC, selected only patients with diffuse brain injury and in the very early phase of therapy, when additional less invasive interventions could have been attempted [97]. In the second trial, 37% of controls also underwent delayed DC as salvage procedure, which produced a significant crossover between the study groups [98].

#### What is new

Despite the Brain Trauma Foundation recommends against bi-frontal DC [99], large DC, either unilateral or bilateral, might still be considered in some patients, such as those with neurological deterioration between admission and re-examination (i.e. secondary brain injury), as this would exclude severe primary injury and brainstem lesions [100, 101]. As such, DC remains a procedure with uncertain benefits on neurological recovery that deserves a multidisciplinary and rational approach, based on clinical trajectories and imaging. As ethical concerns are at stake with this procedure, relatives should be involved in the decision process.

### Clinical dilemmas: outcome prediction after head trauma

#### What is important

The International Mission on Prognosis and Analysis of Clinical Trials (IMPACT) and the Corticosteroid Randomization after Significant Head Injury (CRASH) scores have been developed from large datasets and were externally validated to predict mortality and neurological outcome after traumatic brain injury (TBI) at 6 months [102–104]. Nevertheless, these scores are limited because functional recovery may continue for at least 18 months following TBI in some patients and because of the large heterogeneity in outcome prediction for an individual patient.

#### What is new

Advanced magnetic resonance imaging (MRI)—with the use of susceptibility-weighted-imaging (SWI), diffusion-weighted-imaging (DWI) and high-definition-fibre-tractography (HDFT)—improves outcome prediction, despite the lack of evidence supporting its routine clinical use [105, 106]. MRI diffusion tensor imaging (DTI) and fractional anisotropy (FA) assess white matter integrity and are important in predicting outcome [107, 108]. The Coma Score was developed using MRI-DTI to predict 1-year outcome of patients unresponsive to simple orders after HIBI between day 7 and 45 after initial injury [65]; current ongoing research is evaluating this approach to predict TBI outcome. In a cohort of 105 comatose TBI patients, the area under the curve of the DTI score to predict poor outcome was 0.84 (95% CI: 0.75–0.91) [109].

## Abbreviations

BRASS: Brainstem Reflexes Assessment Sedation Scale
CAM-ICU: Confusion Assessment Method for the ICU
CRASH: Corticosteroid Randomization after Significant Head Injury
CSF: cerebrospinal fluid
CT: computed tomography
DC: decompressive craniectomy
DCI: delayed cerebral ischaemia
DOAC: direct oral anticoagulants
EEG: electro-encephalography
DTI: diffusion tensor imaging
DWI: diffusion-weighted-imaging
ENMG: electroneuromyography
FA: fractional anisotropy
FOUR: Full Outline of Unresponsiveness
GCS: Glasgow Coma Score
HDFT: high-definition-fibre-tractography
HIBI: hypoxic ischaemic brain injury
ICDSC: Intensive Care Delirium Screening Checklist
ICU: intensive care unit
ICU-AW: ICU-acquired weakness
IMPACT: International Mission on Prognosis and Analysis of Clinical Trials
MCA: middle cerebral artery
MRC: Medical Research Council
MRI: magnetic resonance imaging
mRS: modified Rankin Scale
NMDA-R: anti-*N*-methyl-d-aspartate receptor
NSE: neuron-specific enolase
PACNS: primary angits of the central nervous system
PIC: Paris Intensive Care
RASS: Richmond Agitation Sedation Scale
RSE: refractory status epilepticus
SRSE: super-refractory status epilepticus
SWI: susceptibility-weighted-imaging
TCD: transcranial doppler
TOACT: Thrombolysis or anticoagulation for cerebral venous thrombosis
TTM: targeted temperature management
VKA: vitamin K antagonists
